# Multifunctional Immunoadjuvants for Use in Minimalist Nucleic Acid Vaccines

**DOI:** 10.3390/pharmaceutics13050644

**Published:** 2021-05-01

**Authors:** Saed Abbasi, Satoshi Uchida

**Affiliations:** 1 Innovation Center of NanoMedicine, Kawasaki Institute of Industrial Promotion, 3-25-14 Tonomachi, Kawasaki-ku, Kawasaki 210-0821, Japan; 2 Medical Chemistry, Graduate School of Medical Science, Kyoto Prefectural University of Medicine, 1-5 Shimogamohangi-cho, Sakyo-ku, Kyoto 606-0823, Japan

**Keywords:** pDNA, mRNA, subunit vaccine, adjuvant, nonviral vaccine

## Abstract

Subunit vaccines based on antigen-encoding nucleic acids have shown great promise for antigen-specific immunization against cancer and infectious diseases. Vaccines require immunostimulatory adjuvants to activate the innate immune system and trigger specific adaptive immune responses. However, the incorporation of immunoadjuvants into nonviral nucleic acid delivery systems often results in fairly complex structures that are difficult to mass-produce and characterize. In recent years, minimalist approaches have emerged to reduce the number of components used in vaccines. In these approaches, delivery materials, such as lipids and polymers, and/or pDNA/mRNA are designed to simultaneously possess several functionalities of immunostimulatory adjuvants. Such multifunctional immunoadjuvants encode antigens, encapsulate nucleic acids, and control their pharmacokinetic or cellular fate. Herein, we review a diverse class of multifunctional immunoadjuvants in nucleic acid subunit vaccines and provide a detailed description of their mechanisms of adjuvanticity and induction of specific immune responses.

## 1. Introduction

Subunit vaccines have demonstrated considerable efficacy and safety against infectious diseases and cancer in clinical settings [1,2]. Specifically, the introduction of antigens using plasmid DNA (pDNA) and messenger RNA (mRNA) has been shown to be effective in meeting urgent clinical needs during pandemics and for targeting cancer neoantigens due to nucleic acid ease of production, flexible design, and high efficacy [3,4]. However, a nonviral delivery system is an integral part of the pDNA/mRNA-based vaccination. A nonviral vaccine formulation needs to provide all of the following functions: (i) encode at least one antigen; (ii) encapsulate and protect the antigen-encoding nucleic acid; (iii) form a particulate system for favorable pharmacokinetics following administration; (iv) efficient transfection, including cellular internalization, endosomal escape, and nuclear entry if needed; and (v) activation of the innate immune system required to induce protective immunity. Designing nonviral vaccines incorporating all of the abovementioned functions often results in fairly complex structures that contain multiple materials, all working together to induce a desired immune response. Thus, recent trends have shifted towards the development of minimalist vaccines containing the lowest number of materials required to induce a robust immune response with a low level of adverse effects [5].

Minimalizing vaccine complexity allows an easier production and characterization of vaccines, which in turn facilitates the processes needed to gain authorization for deployment and manufacturing in high quantities, which are needed to meet the urgent clinical needs, for example, in the case of pandemics such as the current one. The minimal essential number of components needed to define a minimalist nucleic acid vaccine has not been well-defined, but this can highly vary depending on the type of vaccine and intended application. For example, high levels of safety are needed for vaccines for preventing infectious diseases, which are delivered to a large number of people, including children, the elderly and people with underlying medical conditions. In contrast, a certain level of adverse effects can be tolerated in cancer vaccines, especially in advanced stages, in which the vaccine needs to overcome the immunosuppressive mechanisms of cancers. In all cases, each vaccine should be designed to address these issues using minimal components, to minimize the adverse effects while inducing a desired immunization, in a balanced risk versus benefit equation. Design strategies that address the abovementioned points (i–iv), with regard to the development of nonviral delivery systems for nucleic acid delivery, have been extensively reviewed in previous articles [6,7,8,9,10,11]. Thus, in the present review, we focused on materials that activate the innate immune system (v) while simultaneously meeting other requisites (i–iv) of nucleic acid delivery in nonviral delivery systems as a minimalist approach in vaccine development.

The activation of the innate immune system is a critical step that needs to be carefully orchestrated to induce a balanced immune response and minimize toxicity. Immunoadjuvants, or simply adjuvants, are a diverse class of substances required to initiate or direct an antigen-specific immune response, and their mechanisms of action remain poorly understood [12,13,14]. Adjuvants act either nonspecifically by generating inflammation near the injection site or by activating molecular targets needed to elicit a specific immune cascade [15]. The delivery of adjuvants can be carried out separately from the antigen or coformulated with the antigen in one system. Recent evidence has demonstrated that the codelivery of the adjuvant and antigen in one formulation induces a more robust and durable immunization compared to separate delivery [16,17,18,19,20,21]. For the codelivery of antigen-encoding pDNA/mRNA and adjuvants in one formulation, multifunctional adjuvant materials that simultaneously possess other functionalities, such as the encoding of antigens, packaging of pDNA/mRNA, and contributing to favorable pharmacokinetics and intracellular processing, are preferred as a minimalist approach. For example, cationic lipids and polymers are often designed to induce immunostimulation, package pDNA/mRNA, and facilitate nucleic acid delivery; therefore, their incorporation into vaccines reduces the need for other functional materials. In the following sections, we review the development of pDNA/mRNA nonviral vaccines using multifunctional adjuvant materials, with a detailed description of their mechanism of adjuvanticity and strategies to elicit a specific immune response.

## 2. Functions of Nucleic Acid Vaccines

**Figure 1:**
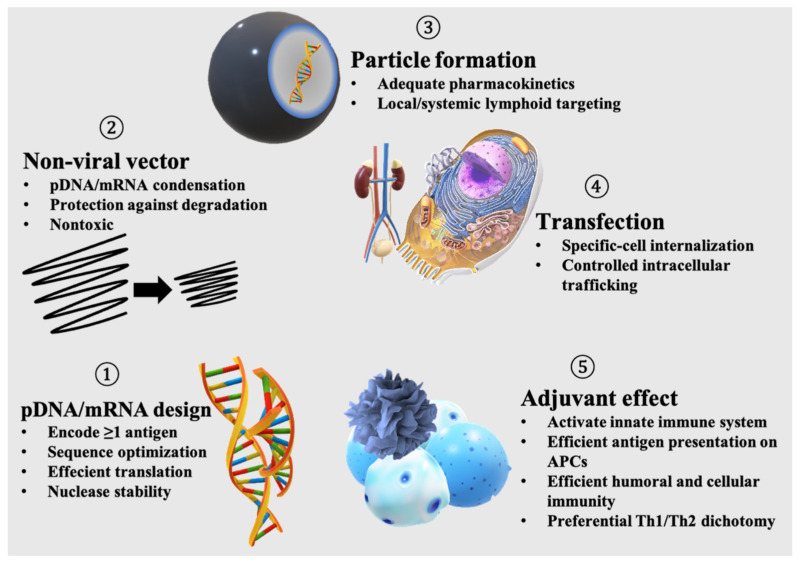
Functions needed for an effective nucleic acid-based subunit vaccine.

Viruses are nanosized carriers of nucleic acids that date back billions of years [22]. Viruses are obligate parasites whose life cycles are only initiated upon the successful transfection of host cells; thus, they are naturally very efficient in gene transfer [23]. Due to longstanding safety concerns and production difficulties associated with the viral vectors used in gene delivery, subunit vaccines enabled by nonviral delivery systems have emerged as a new class of vaccines [1,2,24,25]. Nonetheless, a pDNA/mRNA nonviral vaccine needs to integrate all of the functions encrypted in viruses to induce a potent and desired antigen-specific immunization (Figure 1). In addition, nonviral vaccines should possess adjuvant functionalities, in contrast to viruses that try to escape from the immune system.

### 2.1. Antigen-Encoding

Protein/peptide-based subunit vaccines represent very important and clinically relevant strategies for immunization against infectious diseases, cancer and neurodegenerative diseases such as Alzheimer’s disease [26]. Recently, recombinant spike protein-based COVID-19 vaccines showed promising outcomes in a clinical trial [27]. Alternatively, intracellular delivery of antigens by pDNA/mRNA also represents a promising approach to induce cellular immunity as well as humoral immunity [28]. Both pDNA and mRNA express antigen peptides or proteins using the transcription/translation machinery of the host cells but differ in their protein expression kinetics. The protein expression levels of pDNA rise gradually after transfection and remain constant for days, while mRNA expresses high amounts of protein shortly after transfection, which diminishes after a few days [29]. Enhanced and prolonged protein expression can be obtained via codon modifications; the choice of promoter in pDNA [30]; design optimization of the 5′ cap, polyA tail, and untranslated regions in mRNA; or the use of self-amplifying RNA (replicon), which allows for prolonged protein expression [31,32,33]. For vaccination, pDNA/mRNA has the advantage of encoding multiple antigens. An individualized melanoma FixVac mRNA vaccine encoding four tumor-associated antigens (TAAs) demonstrated efficacy in the interim analysis of a first-in-human, dose-escalation phase I trial in patients with advanced melanoma [34].

### 2.2. Encapsulation of Antigen-Encoding Nucleic Acid

Nucleic acids are large polyanions that are poorly permeable across the plasma membrane. The condensation of nucleic acids in nonviral vectors has been accomplished using various cationic polymers or lipids [35]. pDNA, a double-stranded nucleic acid, and mRNA, a single-stranded nucleic acid, have different condensation behaviors depending on the carrier system. For example, pDNA condenses into rod-shaped structures when complexed with polyethylene glycol (PEG) polycations, while mRNA forms spheres [36,37,38]. In the case of lipid nanoparticles (LNPs), nanosized lipid aggregates complexed with nucleic acids in the core by means of electrostatic interaction, the optimal formulation composition differs between the delivery of pDNA and mRNA. For example, mRNA LNPs show efficient protein expression by using saturated helper lipids, whereas unsaturated helper lipids are preferred in pDNA LNPs [39]. Notably, nonviral vectors should provide nucleic acids with sufficient protection against nuclease degradation, while maintaining a high margin of safety in the biological milieu.

### 2.3. Particulate Formation

Particle size and surface charge play crucial roles in dictating antigen and adjuvant pharmacokinetics and subsequent vaccine efficacy [40]. For intravenous vaccination against cancer, 200–400 nm nanoparticles, such as anionic lipoplexes, are used to target major lymphoid organs, such as the spleen [41]. Following local vaccination via subcutaneous or intramuscular routes, although the particle size effect may vary, particulate formulation of antigens is indispensable to obtain strong immunization compared to soluble antigens. Lynn et al. showed that large polymer particles containing adjuvants of approximately 1 µm in size were efficiently taken up by migratory dendritic cells (DCs) and monocyte-derived antigen presenting cells (APCs), trafficked to draining lymph nodes (dLNs), and induced higher adjuvant activity compared to soluble polymer coils [42]. For the targeting of lymph nodes, Nakamura et al. showed that 30 nm LNPs prepared by microfluidic mixing could penetrate deeper regions inside the lymph nodes, which resulted in a higher uptake by DCs and induced the priming of CD8+ T cells [43]. However, a balanced particle size appears to be required to target the lymph nodes for vaccination. In a previous study, 40 nm-sized liposomes (nanosized vesicles composed of a phospholipid bilayer) were found to be readily transported to the lymph node but exhibited the lowest retention, while larger liposomes, 400 nm or larger, showed poor transport but a higher retention [44]. From an industrial viewpoint, vaccine solutions containing particles <200 nm in size are easier to sterilize by filtration, while those containing larger particles need to be sterilized by other means, such as UV-light irradiation [45].

Furthermore, particulate formations provide a platform to coencapsulate the adjuvant and antigen/antigen-encoding nucleic acids in one carrier, which often results in higher antigen presentation efficiency in DCs and limits the distribution of the adjuvant, thereby minimizing systemic toxicity. Nanodiscs with the capability to tether both neo-epitope antigens and immunostimulatory adjuvant CpG oligodeoxynucleotide (CpG ODN) drastically improved the delivery of both components into dLN, sustained antigen presentation on DCs, and induced the efficient cross-priming of T-cells [18]. Similarly, the conjugation of antigen peptides together with CpG ODN onto a pH-sensitive polymer nanoparticle enhanced antigen cross-presentation, compared to the delivery of a physical mixture of antigen and adjuvant [17]. Owing to their negative charge, pDNA/mRNA enables a universal encapsulation method using cationic materials, which possess adjuvant activity, as will be discussed in Section 3.1.1 and Section 3.1.2. Therefore, multifunctional adjuvants that can complex pDNA/mRNA and form particulate matter by supramolecular assembly represent an important class of materials that enable minimalist vaccines.

### 2.4. Transfection

Nucleic acids must be internalized inside the cell. Following cellular uptake, mRNA is trafficked to the cytosol, while pDNA is trafficked to the nucleus. Nonviral delivery vectors can be internalized by cells via nonspecific endocytic pathways, such as micropinocytosis, clathrin- or caveolae-mediated pathways, or specific endocytosis if a receptor-specific ligand is used [46]. Vectors equipped with endosomal escape functionalities are often characterized by higher protein expression efficiency [47,48,49]. In the cytosol, mRNA needs to remain sufficiently stable to maximize translational activity. In contrast, pDNA has to translocate into the nucleus during mitosis in dividing cells or penetrate through the nuclear pore complex in nondividing cells. Various strategies have been proposed to control the intracellular trafficking of pDNA, including chemical modification by attaching nuclear localization signals (NLSs) derived from viruses, or the use of delivery devices, such as iontophoresis, electroporation, sonophoresis, microneedles, and jet injection [50,51,52].

### 2.5. Adjuvanticity

**Figure 2:**
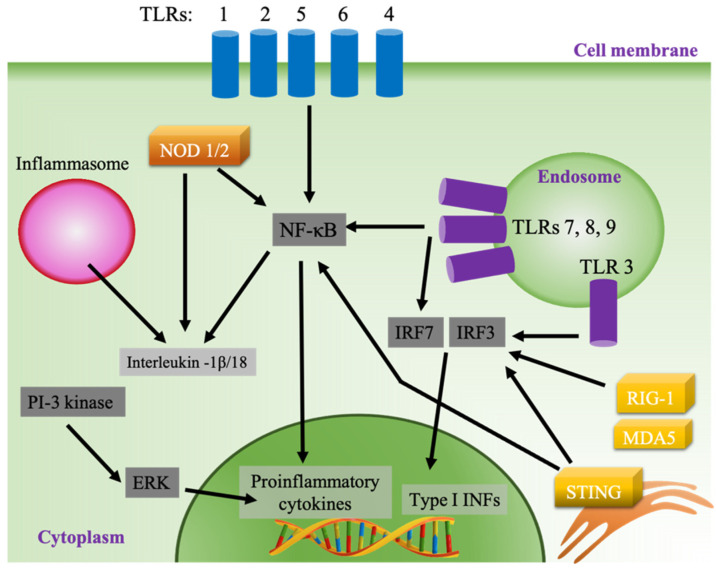
Pathways of innate immune system activation.

Vaccines act by inducing an adaptive immune response to protect against a pathogen by mimicking the naturally occurring infection without causing disease [53]. In contrast to viral infection, subunit vaccines lack the ability to activate the innate immune system, which is needed to successfully recognize and present antigens to adaptive immune cells. The inclusion of adjuvants in subunit vaccines has allowed for robust immunization against pathogen-specific antigens [54]. Synthetic adjuvants act by causing local inflammation, mainly by activating pattern recognition receptors (PRRs), which are expressed in different cellular compartments in innate immune cells [13,55,56]. One of the main classes of PRRs is Toll-like receptors (TLRs), which are expressed on the cell surface (TLRs 1, 2, 4, 5, and 6) or in the endosome (TLRs 3, 7, 8, and 9) (Figure 2). The activation of surface TLRs by pathogen-associated molecular patterns (PAMPs) subsequently activates the production of proinflammatory cytokine via the nuclear factor kappa-light-chain-enhancer of activated B cells (NF-κB) pathway through activation of myeloid differentiation primary response protein 88 (MyD88) [57,58]. Contrastingly, the activation of MyD88 when endosomal TLRs 7, 8 and 9 are stimulated results in the activation of interferon-regulatory factor 3 (IRF 3) and the production of type I interferons (IFNs), while stimulated TLR 3 produces type I INFs through the MyD88-independent pathway using TIR-domain-containing adapter-inducing interferon-β (TRIF) as its adaptor. Type I INFs are potent activators of lymphocytes, especially CD8+ T cells and cytotoxic T lymphocytes (CTL) [59,60]. In addition, endosomal TLRs 7, 8 and 9 activate NF-κB through a MyD88-dependent pathway (Figure 2). Cytosolic PRR represents another class of sensors that can be targeted by adjuvants, such as the retinoic acid-inducible gene I (RIG-I), RIG-I-like receptors such as melanoma differentiation-associated protein 5 (MDA5), and the endoplasmic stimulator of interferon genes (STING) pathways [61,62]. Adjuvants can also stimulate the secretion of chemokines and cytokines by activating other inflammatory pathways, such as the inflammasome and the extracellular-signal-regulated kinase (ERK) pathway (Figure 2) [63,64].

Adjuvants create a proinflammatory environment in APCs and other cells at the site of injection initiated by the secretion of chemokines and cytokines, including chemoattractants, such as CCL2, CCL3, CCL3, and CXCL8 [14,15]. Then, professional antigen-presenting innate immune cells, such as DCs, macrophages, and monocytes, are recruited, activated, and matured. Mature APCs carrying costimulatory molecules, such as CD80/CD86, and presenting antigen epitopes on major histocompatibility complex (MHC class 1/2) then interact with T and B lymphocytes to produce antigen-specific immunization. Depending on the delivery system, adjuvants can be directly targeted to lymphoid organs where APCs and lymphocytes exist in close proximity, or activate tissue-resident or infiltrated APCs present at the side of local injection, which then migrate to draining lymph nodes to interact with lymphocytes [65].

Adjuvants can also skew the immune response depending on their mode of T helper (Th) cell activation and the types of cytokines released to mediate Th1, Th2, or other Th cell expansion [66]. Alum adjuvants (mixture of aluminum hydroxide and magnesium hydroxide) are biased towards a Th2 immune response as a result of IL-4 secretion during Th cell differentiation. The Th2 response mediates acute humoral immunity needed to eliminate extracellular pathogens, such as parasites, by secreting antibodies and activating neutrophils and eosinophils [67]. In contrast, monophosphoryl lipid A (MPLA) is associated with an immune response tilted towards the Th1 type as a result of IFN-γ, IL-12, and tissue necrosis factor (TNF-α) during Th cell differentiation. The Th1 response is involved in the development of cellular immunity against intracellular pathogens, such as viruses, and for the killing of cancer cells [68]. IFN-γ secreted by activated CD4+ Th1 cells also regulates the production of opsonizing antibodies, such as immunoglobulin IgG2a, to eliminate pathogens by phagocytosis [69]. However, many synthetic adjuvants induce a balanced Th1/Th2 response, which is needed to develop both humoral and cell-mediated adaptive immunity. Additionally, the codelivery of multiple adjuvants has been found to be synergistic if these adjuvants activate different pathways, and antagonistic if the same pathways are targeted using more than one adjuvant at a time [70]. In the next section, we summarize the research efforts undertaken in developing multifunctional adjuvants to enable the development of minimalist pDNA/mRNA vaccines (Table 1).

**Table 1:** List of multifunctional immunoadjuvants for incorporation in nucleic acid vaccines.

Multifunctional Adjuvant	Subclass	Adjuvant Mechanism	* Other Functions	Reference
Natural Lipids	Squalene and α-tocopherol	Macrophage and inflammasome stimulation, antigen uptake	1, 2	[71,72,73]
	LPS	TLR4 activation	1	[74]
	Saponin	Antigen uptake, costimulatory signal adduct formation	1, 2	[75,76,77]
Synthetic Lipids	MPLA	TLR4 activation	1	[77,78,79]
	αGalCer	NKT activation	1	[80,81,82]
	CCL-34	TL4 activation, autophagy	1, 2	[83]
	TDB	TLRs 2,3,4,7-independent (Myd88-dependent), Syk–Card9 pathway	1, 2	[84]
	Stearyl-KALA	STING/TBK1and inflammasome activation	1, 2	[85,86]
	Pam3	TLR 1/2 activation	1, 2	[87]
	Quaternary ammonium lipids	ERK pathway (NF_k_B-independent), TLR 7/9 activation, unknown	1, 2, 3	[88,89]
	Ionizable lipids	Similar to cationic lipids, unknown	1, 2, 3	[90,91,92,93,94]
	Heterocyclic lipids	STING pathway activation	1, 2, 3	[95]
	ssPALME	STING pathway activation	1, 2, 3	[96]
	C1 lipid	TLR 4 activation	1, 2, 3	[97]
Natural and semi-synthetic polymers	Protamine	TLR 7/8 activation	1, 2, 3	[98,99]
	Chitosan	STING pathway activation	1, 2, 3	[100,101,102]
	Dextran sulfate	Uptake by lymphocytes	1, 2	[103]
	Cyclodextrins	Lipid raft formation	1	[104,105]
Synthetic polymers	PEI	TLR4/5 activation, DNA leakage (IRF 3-dependent)	1, 2, 3	[105,106]
	Poly(l-Lysine)	TLR4 activation	1, 2, 3	[72]
	PEG-b-PC7a	STING pathway activation	1	[107]
	PLG-CTAB	Efficient uptake by APC	1,2,3	[108]
	PP TLR7/8a	TLR 7/8 activation	1	[42]
	Polyphosphazene	TLRs interference, chemokine release	1, 2	[109,110,111]
Single-stranded nucleic acids	Unmodified mRNA	TLR 3/7/8 activation	4	[112,113,114]
	5′cap-modified mRNA	NF-κB signaling	4	[115]
Double-stranded nucleic acids	CpG-modified plasmid	TLR 9 activation	4	[116,117]
	Adjuvant-expressing pDNA	RIG-I or TLR9 activation	4	[118,119]
	CpG ODN nanogel	TLR 9 activation	1	[120,121]
	Poly I:C and derivatives	TLR3 activation	1, 2	[122]
	hybridized mRNA	RIG-I and TLR3 activation	4, 1	[123]
	Self-amplifying RNA	TLRs 3/7/8, RIG-I and MDA5 activation	4	[124]

* 1: Particle formation by supramolecular assembly, 2: transfection (cellular uptake, endosomal escape or nuclear entry); 3: complexation with pDNA/mRNA, 4: antigen-encoding. Abbreviations: LPS: lipopolysaccharide; MPLA: monophosphoryl Lipid A; αGalCer: alpha-Galactosylceramide; TDB: trehalose 6-behenate; PEI: polyethylenimine.

## 3. Multifunctional Immunoadjuvants

### 3.1. Adjuvants Associated with Delivery System

**Figure 3:**
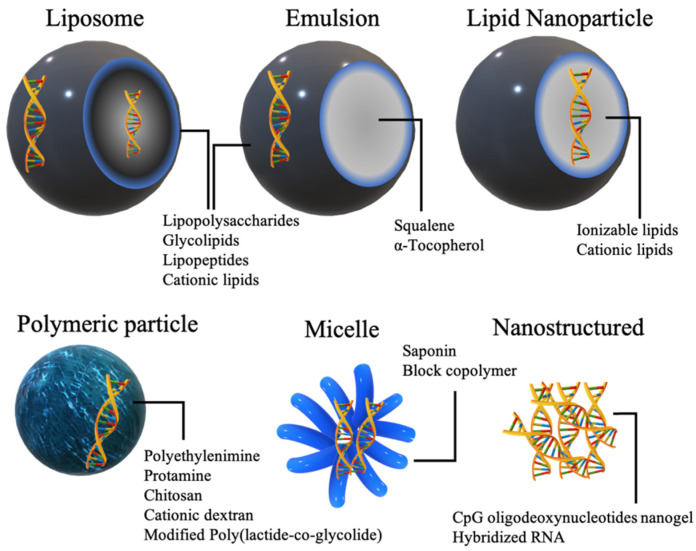
Commonly used nonviral delivery systems for the delivery of adjuvants in nucleic acid vaccines.

Immunoadjuvant materials can be incorporated in a wide range of nonviral nucleic acid delivery systems. Depending on their psychochemical natures, adjuvants can be embedded on the exterior of particles, in the interior or both (Figure 3).

#### 3.1.1. Lipids and Related Structures

##### Naturally Occurring Lipids

- Squalene and tocopherols

Squalene and tocopherols represent the major unsaponifiable fraction of lipids in food [125]. Squalene is also ubiquitously distributed in all human tissues and plays a key role in steroid biosynthesis and free radical scavenging [126,127]. α-Tocopherol, the most abundant form of vitamin E, is also an important antioxidant that is obtained from food or dietary supplements [128]. Driven by their oily natures, squalene and α-tocopherol have long been used in the formulation of oil-in-water (*o*/*w*) emulsion-based adjuvants for vaccination. MF59 by Novartis is a well-established adjuvant formulation of squalene emulsified by the hydrophilic and hydrophobic surfactants Tween 80 and Span 85, respectively, are used in seasonal influenza vaccines and were used on children during the 2009 H1N1 pandemic [129]. MF59 has been shown to induce an immune response independent of the type I interferon (IFN) pathway by inducing proinflammatory chemokines, such as CXCL10, and cytokines that promote the differentiation and recruitment of monocytes, macrophages, and granulocytes [130]. In addition, MF59 assisted in antigen uptake and presentation on the surface of immune cells [131]. The inclusion of α-tocopherol in squalene emulsions enhanced the overall immunostimulatory function, such as in the case of AS03 by GlaxoSmithKline, which allowed for the induction of higher H1N1 antibody titers with a reduced antigen dose when deployed in 2009 [129].

Further modification of these emulsions was carried out to allow complexation with antigen-encoding pDNA or mRNA. A pDNA encoding HIV-1 pCMVp55 gag DNA was adsorbed on the surface of a cationic DOTAP-modified MF59 emulsion by electrostatic interaction, and induced higher antibody titers compared to naked pDNA in mice and rabbits [71]. Although MF59 adjuvants used in protein vaccination usually tilt towards a Th2 response, DNA adsorbed on cationic MF59 emulsion showed a desired Th1 response, as evidenced by a higher production of the IgG2a isotype than IgG1. The same cationic emulsion formulation was used to adsorb a 9 kb single-stranded self-amplifying (SAM) RNA, which resulted in an increase in particle size from 101 to 129 nm following the addition of RNA [73]. The vaccine was tested using three model antigens from pathogens that represent unmet medical needs: the fusion (F) glycoprotein of respiratory syncytial virus (RSV), envelope glycoprotein B (gB), and a fusion protein (pp65–IE1) of phosphoprotein 65 (pp65), immediate early protein 1 (IE-1) from human cytomegalovirus (hCMV), and gp140 envelope glycoprotein (env) of human immunodeficiency virus (HIV). Both antibody and T-cell immunity were efficiently induced following intramuscular injection in mice, rats, and rhesus macaques, which was comparable in magnitude to the immunity obtained using RNA packaged in alphavirus-based viral replicon particles (VRPs). However, in the same study, the immunogenicity of SAM RNA was found to be mostly dependent on the presence of the cationic charge of DOTAP in modified MF59 emulsion, since SAM RNA mixed with MF59 did not show a significant increase in IgG titers compared to SAM RNA dispersed in saline, while the presence of DOTAP boosted IgG production.

b. Glycosides

Lipopolysaccharides (LPSs), also known as endotoxins, are constituents of the outer monolayer of the outer membrane of Gram-negative bacteria. LPS shares a common structural hierarchy consisting of a lipid A backbone, a middle oligosaccharide region, and an outer *O*-polysaccharide moiety [132]. LPSs exist in a myriad of structural varieties depending on the bacterial species, differing in their potency of immune activation, mechanisms, and toxicities [133]. Early studies showed that the incorporation of LPS W from *S. abortus equi* in egg-lecithin liposomes resulted in a higher antibody production against human serum albumin (HSA) as a model antigen when injected intravenously into rabbits [74]. However, due to their severe systemic toxicity, such as sepsis and septic shock, chemically degraded forms of LPS containing lipid A backbones have been used as vaccine adjuvants instead (see section below).

Saponins are plant-derived amphipathic molecules with mild surfactant activity composed of a lipophilic triterpenoid flanked by one or more oligosaccharides [134]. Saponin extracted from *Quillaja saponaria* Molina (Quil A) was used to form 35 nm micelles of viral membrane proteins based on hydrophobic interactions in the core [135]. These particles, named iscoms, stimulate the production of antibodies against influenza, measles, and rabies in rodents. The saponin-based Matrix-M™ platform patented by Novavax has been tested in humans as an adjuvant for vaccination using full-length, prefusion SARS-CoV-2 spike protein [27]. The addition of Quil A has been reported in veterinary vaccines based on pDNA/chitosan, which resulted in enhanced immunogenicity and formed more defined, less aggregated sub-100 nm particles, as observed by transmission electron microscopy (TEM) [75]. Saponin and chitosan self-assembled nanoparticles could also protect the adsorbed pDNA from enzymatic degradation [76]. The mechanism of adjuvanticity induced by saponins may be attributed to its direct interaction with lectins on the surface of lymphocytes, facilitating antigen uptake and presentation on APCs. It has also been reported that saponins can form imine bonds between the amine groups of the T cell receptor and the aldehyde group of the triterpene moiety saponin, delivering a costimulatory signal required for T cell activation [136,137,138].

##### Synthetic and Semisynthetic Lipids

- Glycolipids

LPS subjected to hydrolysis is converted to a mixture of polysaccharide side chains and acylated diglucosamines [139]. Monophosphorylated lipid A products, commonly known as MPLA, represent one of the most studied glycolipid adjuvants both in animal models and clinically. MPLA retains the immunostimulatory effect of LPS or lipid A, but with much less pyrogenicity [140]. MPLA activates TLR4 on the cell surface extracellularly, or inside the endosome when taken up by endocytosis and activates an immune cascade, leading to the secretion of cytokines including TNF-α [141]. MPLA has been formulated in numerous lipid-based vaccines, such as liposomes, emulsions, and lipid-based nanoparticles, in animal models and humans [142,143,144]. One GSK adjuvant formulation, AS01, which is a liposome containing saponin and MPLA, has been designed to strengthen the CD8+ response and obtain approval for human use in malaria and herpes zoster virus subunit vaccines [77]. MPLA adjuvant formulations can be further optimized to incorporate antigen-encoding nucleic acids. Solid lipid nanoparticles (SLNs), nanosized emulsions containing solid oil cores at room and body temperatures (Figure 3), decorated with MPLA as an adjuvant surfactant and DC cholesterol as a cationic surfactant, were used to adsorb pDNA on the surface to protect pDNA from enzymatic degradation by strong electrostatic attractions [78]. MPLA incorporated into nucleoside-modified mRNA lipoplexes based on DOTAP/cholesterol helped to restore the immune response to levels comparable to those of unmodified mRNA lipoplexes [79]. Unmodified mRNA is usually characterized by self-adjuvating activity as we will discuss in Section 3.2.1. Interestingly, nucleoside-modified mRNA MPLA-lipoplexes exhibited a higher antigen protein expression from the unmodified mRNA, which was attributed to the reduction in type I IFN stimulation as a result of chemical modification, while the capacity to induce cytotoxic T cell response was maintained. Type I IFNs produced by cells after stimulation by unmodified mRNA could have inhibitory effects on antigen expression through indirect mechanisms [79].

Trehalose 6,6′-dibehenate (TDB), a synthetic glycolipid analog of trehalose 6,6′-dimycolate (TDM, also known as cord factor), is a constituent of the mycobacterial cell wall [145]. TBD, coformulated with cationic lipid DDA in liposomes (CAF01), enhanced the immunostimulatory adjuvant effect of this cationic formulation and also helped to further increase the storage stability of the liposomes at both 4 and 25 °C [146]. The self-replicating RNA genome of Chikungunya virus adsorbed on CAF01 liposomes was prepared by vortex mixing of RNA and CAF01. It was found to protect against Chikungunya virus disease in immunocompetent mice when challenged at the site of injection in the footpad, but did not develop systemic protection [84]. The mechanism by which CAF01 stimulates INF secretion was found to be TLRs 2-, 3-, 4-, and 7-independent, as evidenced by the failure to cause a reduced immune activation effect in these specific TLR knockout mice, whereas the response is partly reduced in MyD88-deficient mice, indicating the involvement of TLR other than TLRs 2, 3, 4, and 7, or other pathways of INF secretion [147]. DDA/TDB liposomes induce higher antigen uptake and maturation of DCs more efficiently than DOTAP or DSPC liposomes [148]. TDB alone activates APCs via Syk–Card9–Bcl10–Malt1 independently from the TLR pathway [149].

A glycolipid designed with specific TLR4 agonistic specificity, named CCL-34, exhibited an interesting mechanism of action, in which it promoted antigen processing and presentation on macrophages by inducing autophagy through an NF-κB-dependent pathway [83]. CCL-34 can be formulated into liposomes or O/W emulsions as vaccine adjuvant formulations, potentially incorporating pDNA/mRNA into cationic formulations.

Alpha-galactosylceramide (α-GC) is a marine-sponge-derived synthetic glycolipid with both direct and indirect immune responses [150,151]. When α-GC is taken up by antigen-presenting cells, it can be found on the surface by CD1d molecules, activating natural killer T (NKT) cells to release large amounts of interferon-γ. Taking advantage of its amphipathic nature, α-GC was embedded in liposomes modified with the cell-penetrating peptide octaraginine [80,81], resulting in the expansion of NK cells and reduced melanoma-induced lung metastases in mice [80]. The mRNA cancer vaccine was prepared by encapsulating the mRNA/poly-(β-amino ester) core in a liposome composed of multivalent cationic lipids and α-GC. Interestingly, the delivery of mRNA liposomes with free α-GC induced a lower IgG2c/IgG1 antibody ratio compared to codelivery in the same liposome, indicating that liposomal mRNA/α-GC alters the Th1/Th2 balance toward an increased Th1 response [82].

b. Lipopeptides

Lipopeptides represent another class of adjuvants that can be incorporated into lipid-based vaccine formulations. A stearylated KALA peptide embedded in a liposomal vaccine containing protamine-complexed CpG-free pDNA showed strong immune activation and resulted in strong prophylactic and therapeutic antitumor activity when used for the ex vivo transfection of bone marrow-derived dendritic cells (BMDCs) in mice [85,86]. The incorporation of stearylated KALA induced higher transfection into BMDCs, even higher than stearylated R8, which is a common cell-penetrating peptide used for nucleic acid transfection. Once inside the cytosol, the protamine/pDNA core most likely translocates into the nucleus, guided by NLS on protamine. This advantage of KALA was attributed to the amino acid sequence and the α-helical conformation of KALA at physiological pH, both of which are essential for the dual function of transfection and the adjuvant effect [85,86].

Tri-palmitoyl-*S*-glyceryl cysteine linked to the pentapeptide (PAM3CSK4; Pam3) is a lipid that activates TLR 1 and 2 [152]. Pam3 incorporated into mRNA LNP by microfluidic mixing achieved high levels of antigen-specific CD8+ T cell expansion and induced a strong antitumor effect in mice [87]. The incorporation of Pam3 into mRNA LNP synergized its adjuvant activity, due to the involvement of immune stimulation triggered by distinct subclasses of TLRs.

c. Cationic lipids

Liposomes composed of zwitterionic phospholipids as immunological adjuvants were first proposed several decades ago by Allison and Gregoriadis [153]. Cationic lipids were initially synthesized and used for the transfection of pDNA into cultured cells in a process known as lipofection, as introduced by Felgner et al. [154]. DOTMA, DOTAP, and DDA (or the bromide salt DDAB), synthetic cationic lipids based on quaternary ammonium groups, are the most characterized cationic lipids and have been extensively studied for nucleic acid delivery both in vitro and in vivo. In particular, a number of liposomal formulations and lipoplexes utilizing these cationic lipids have been described for vaccination, in which the cationic lipid contributes to nucleic acid complexation, transfection, and the stimulation of an immune response [90,155,156]. Ligand-based cationic liposomes for lymphoid targeting have also been proposed. PEG-biotin-modified DDA/TBD liposomes were efficiently entrapped in draining lymph nodes following the administration of avidin into mice [91]. Unfortunately, due to cytotoxicity and their effects on cellular membranes, their use is limited in vivo [92]. Although shielding the cationic charge was achieved by grafting the liposome surface with PEG, PEGylation compromised gene transfection efficiency, shifting the focus of research to ionizable lipids [93]. However, Kranz et al. recently reported on the effective use of DOTMA or DOTAP coformulated with helper lipids (DOPE or cholesterol) in mRNA lipoplexes for cancer vaccination [94]. This formulation requires only a small amount of cationic lipid to complex antigen-encoding mRNA, giving the lipoplex an overall negative zeta potential that is needed for spleen targeting [94]. This system has also been tested in clinical trials against advanced melanoma [34].

Different chemical structures of cationic lipids adopt different morphologies in various delivery systems, affecting their transfection [92] and potentially their mechanisms of immune activation. The adjuvant effect of cationic lipids appears to be pleiotropic and involves multiple pathways of immune stimulation. A pioneering work of the Huang group investigated the mechanism of the adjuvanticity of cationic liposomes in mouse BMDCs. They found that DOTAP stimulates NF_k_B-independent proinflammatory cytokines by activating phosphatidylinositol 3-kinases (PI3-kinase)/ERK [89]. However, another study reported that DOTAP induces type I IFN secretion through the involvement of Myd88, but not the TRIF or STING pathways [88]. More specifically, DOTAP was found to directly stimulate TLRs 7 and 9, presumably through direct perturbation of the endosomal membrane following particle uptake [88]. Nonetheless, the immunomodulatory mechanisms of cationic lipids appear to be broad and highly conserved in the animal and plant kingdoms [157].

d. Ionizable lipids

Ionizable lipids, also known as pH-sensitive lipids, represent the most clinically relevant class of transfection reagents. The approval of the first systemic RNAi therapy, Onpattro (Patisiran), for the treatment of polyneuropathy in patients with hereditary transthyretin-mediated amyloidosis by the US FDA in 2018 was a breakthrough in the field of nonviral drug delivery [158]. Onpattro utilizes LNP delivery technology based on the pKa-optimized ionizable lipid MC3, which enables hepatocyte transfection following intravenous administration [159]. Advances in nucleic acid delivery using LNPs in recent decades paved the way for another breakthrough in 2020—namely, the emergency use authorization of two mRNA-LNP vaccines against the SARS-CoV-2 pandemic: mRNA-1273 by Moderna and BNT162b2 by BioNTech/Pfizer [160,161]. Both vaccines utilize ionizable lipids (ALC-0315 in BNT162b2 and SM-102 in mRNA-1273), and showed strong antibody and T cell-mediated immunity and >90% efficacy in the in-term analysis of phase III clinical trials [162,163]. Ionizable lipids are highly multifunctional, as they activate the immune system, contribute to nucleic acid complexation, facilitate endosomal escape, and form nanoparticles with the help of other lipids based on self-assembly. Ionizable lipids are continuously optimized for their use in vaccination by enhancing their tolerability and accelerating their metabolism and excretion from the body [164,165]. Detailed descriptions of the structure of ionizable lipids to the mechanism of the immunostimulatory effect are largely lacking; however, since they hold a cationic charge inside the endo/lysosomal compartment, their mechanism of action may be similar to that of permanently cationic lipids.

Increasingly, research on the combination of ionizable lipids in LNPs for the induction of a controlled immune response is underway. A recent study reported on the use of the forgotten commercially available ionizable lipid DODAP, and succeeded in obtaining strong tumor immunization by targeting pDNA to the spleen in mice [166]. DODAP LNPs were mainly distributed to APCs, such as macrophages, DCs, and B cells, with the highest fraction delivered to B cells. Encapsulating SAM RNA in the interior of LNPs based on ionizable lipid C12-200 protected SAM RNA against RNase degradation, and induced a strong antibody response in mice after immunization using SAM RNA encoding for HIV-1 Env gp-140. Interestingly, RNase protection and antibody production were considerably reduced when the SAM RNA was adsorbed on the surface of LNP, which was in contrast to LNPs based on the cationic lipid DDA and DOTAP, which induced both RNase protection and antibody production in comparable levels when the SAM RNA was encapsulated on the interior or exterior of LNPs [167].

**Figure 4:**
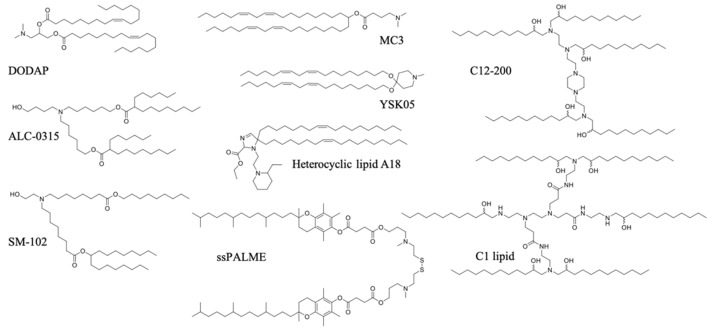
Ionizable lipids used in pDNA/mRNA vaccines.

The rational design and screening of ionizable lipids have also enabled the discovery of lipids that specifically activate the immune system. For example, the attachment of the vitamin E scaffold to a proton-activated lipid-like material was found to activate the STING pathway [96]. This was attributed to the slower rate of pDNA decapsulation from the LNP due to the increased hydrophobicity of the vitamin E scaffold, giving pDNA more time to remain encapsulated without degrading in the acidic endo/lysosome, until its release into the cytosol, where innate immune sensors exist. Therefore, the entire pDNA/LNP system served as an adjuvant. A three-dimensional combinatorial screening of over 1000 lipids revealed the structural features needed for robust STING activation for mRNA delivery. Ionizable lipid-like materials containing a cyclic amine head group, dihydroimidazole linker, and an unsaturated lipid tail were able to inhibit tumor growth and prolong survival in melanoma and human papillomavirus E7 in in vivo tumor mice models [95]. A minimalist mRNA vaccine was formulated using C1 lipid-like ionizable material, which was found to mainly activate TLR4, as evidenced by the abolished antitumor effect in *Tlr4^−/−^* mice. The C1 LNP mRNA nanovaccine, encoding tumor neoantigens, exhibited significant in vivo efficacy in both tumor prevention and therapeutic settings without inducing obvious systemic toxicity [97]. A list of ionizable lipids commonly used in pDNA/mRNA vaccine delivery are shown in Figure 4.

#### 3.1.2. Polymers

##### Natural and Semisynthetic Polymers

- Cationic peptides

Although cationic lipids do not exist in nature, cationic polymers are abundant [168]. Protamine is an arginine-rich peptide that binds DNA in the sperm of all vertebrates [169]. Pharmaceutical grade protamine is approved for human use as an antidote against the anticoagulant heparin, and for other applications, including binding insulin in sustained-release formulations [170]. For drug delivery, protamine has long been used for the condensation of oligonucleotides, pDNA, and mRNA, resulting in solid nanoparticles in the range of 100–200 nm [171]. Protamine has attracted significant attention for use in gene delivery due to its cell-penetrating and nucleus-targeting properties [172]. PEG-PLA nanoparticles functionalized with low molecular weight protamine have been reported to cross the blood–brain barrier in mice following intranasal administration [173]. A highly minimalist vaccine based on antigen-encoding mRNA and protamine, by CureVac, showed safety and promising efficacy in human phase 1/2 clinical trials, in which the patients received intradermal vaccination against metastatic melanoma [98]. Protamine-stabilized mRNA prophylactic vaccines have also been tested in humans against infectious diseases, such as the rabies virus in phase 1 clinical trials [99]. The vaccine showed good tolerability but was only efficacious when administered using a needle-free injection device. The mechanism of the adjuvanticity of protamine-RNA was reported to be related to TLR7/8 activation and was found to be strictly contingent upon complexation with mRNA [174]. Human PBMCs exposed to either mRNA or protamine alone did not produce any measurable cytokine production, while a combination of both resulted in a significant production of IFN-α, regardless of the protamine source or its salt form [174].

b. Carbohydrates

Chitosan is prepared by the deacetylation of chitin extracted from marine crustaceans [175]. Chitosan is widely utilized in the pharmaceutical industry as an excipient, and the presence of both hydroxyl and amine groups in chitosan makes it a highly versatile polymer for use in drug binding and functionalization [176,177]. Owing to its ability to complex with nucleic acids and induce cellular internalization based on charge interactions, chitosan has been widely used for studies involving gene transfection [178,179,180]. DNA vaccines prepared by the coacervation of pDNA and chitosan into nanoparticles have been used for mucosal immunization against the Coxsackievirus B3 virus, and to induce both mucosal and humoral immunity against *Trueperella pyogenes* infections [100,101]. As a vaccine adjuvant, chitosan induces the activation of cGAS-STING by mitochondrial stress and DNA release, leading to the type I INF-mediated activation of dendritic cells and the subsequent activation of cellular immunity in mice [102]. In addition, chitosan, but not chitin, can induce the activation of the NLRP3 inflammasome in a phagocytosis-dependent manner [102,181]. Several chitosan-based vaccines have been studied in human clinical trials, as reviewed by Vasiliev [182].

Dextran sulfate (DS) is an anionic polysaccharide with strong antithrombogenic properties [183]. DS is a potent adjuvant for cell-mediated delayed-type hypersensitivity immune responses in both mice and guinea pigs [184]. Lymphocytes present receptors for sulphated polysaccharides, such as dextran sulfate and carrageenan; therefore, particle modification with dextran sulfate might induce an immune response via the modulation of specific immune cells [185]. Although DS does not form complexes with nucleic acids, it can be used in combination with cationic polymers to mitigate their charge-induced cytotoxicity [103]. Cationic derivatives of dextran, such as diethylaminoethyl dextran (DEAE-D), have been studied for DNA condensation due to their low cost [186]. Their combination with an inactivated typhoid vaccine enabled dose reduction as a result of their strong adjuvant effect in mice [187]. However, their use in humans has been limited due to their induction of sarcoma formation at vaccine injection sites in mice [188].

Cyclodextrins are highly versatile oligosaccharides produced from starch by enzymatic reactions [189]. Cyclodextrins participate in host‒guest-type interactions with various small molecules, giving them advantages for use in drug delivery [190]. These classes of materials are unionized and therefore lack the capacity to bind nucleic acids by electrostatic interactions. Thus, their use as carriers for nucleic acid vaccine formulation is not applicable. The hydroxyl groups of cyclodextrin can be further modified to cationic species, allowing for complexation with nucleic acids. pDNA complexed with polycationic amphiphilic cyclodextrin nanoparticles showed efficient pDNA transfection by clathrin-dependent pathways, although a fraction was taken up by a clathrin-independent pathway [191]. mRNA vaccines based on polyethylene imine-conjugated cyclodextrin were prepared at various nitrogen/phosphate (N/P) ratios ranging from 4 to 24; however, the highest transfection efficiency was observed at an N/P ratio of 16 [105]. Ovalbumin-encoding mRNA vaccine boosted antibody titers following different routes of immunization, with a prominent Th1 immune response as opposed to antigen protein delivery [105]. Cyclodextrin has been reported to have an interesting adjuvant activity by interfering with lipid rafts on the cell membrane of dendritic cells, causing their maturation [104]. This was confirmed using filipin, a lipid raft inhibitor, which attenuated hydroxypropyl-β-cyclodextrin-induced DC maturation, cytokine expression, and T lymphocyte-stimulating activities in vitro. In mice, ovalbumin-specific antibodies were markedly elevated following vaccination with ovalbumin and cyclodextrin as adjuvant [104].

##### Synthetic Polymers

- Cationic polymers

Polyethylene imine (PEI) exists in different architectures, including linear and branched structures of varying lengths [192,193]. PEI holds secondary, tertiary, and primary amine groups in its backbone, giving PEI different ionization degrees depending on its structure at different pH values. Although PEI represents a gold standard for DNA condensation in polymer-based transfection methods, its use is limited by its cytotoxicity [194]. PEI polyplexes are readily taken up by endocytic pathways and exhibit endosomal escape properties via a proton-sponge effect [195,196]. The PEI mechanisms of activation of the immune system are broad. Cationic polymers, including PEI, polylysine, and cationic dextran, were found to activate TLR4 in vitro, which is the receptor of LPS [72,197]. Polymers with larger molecular weights induce more significant activation [198]. In mice, PEI induced a strong activation of TLR5, as evidenced by the production of hallmark TLR5-inducible cytokines, which were absent in Tlr5-/- knockout mice [199,200]. Interestingly, PEI-associated cellular toxicity plays a key role in immune system activation. dsDNA released from apoptotic cells appears to induce IFN-γ production by the activation of intracellular DNA sensors, a mechanism similar to that of aluminum-based adjuvants [106]. Additionally, the perturbation of plasma endo/lysosomal membranes by PEI could be the reason for the observed activation of the Nlrp3 inflammasome and IL-1B production in mice [106]. Due to its toxicity, the use of PEI is generally restricted as a reagent for in vitro/in vivo transfection, which impedes its development into clinical trials [201]. However, a phase 1 clinical trial study design using PEI-condensed fusion DNA encoding a patient-specific antigen for the treatment of B-cell non-Hodgkin’s lymphoma has been reported [202].

Poly (amino acid)-based cationic polymers have also been used for vaccination. An amphiphilic block copolymer composed of poly-l-lysine as a hydrophilic and cationic segment and a hydrophobic segment grafted with phenyl moieties was used as an emulsifier to prepare squalene nanoemulsions. Following intramuscular injection in mice, the adjuvant system induced a Th1 immune response, presumably as a result of TLR4 activation by the cationic poly-l-lysine, and a Th2 immune response induced by squalene. The cationic surfaces of these nanoemulsions allowed for efficient internalization by APCs and the adsorption of antigens by electrostatic interactions [72]. A minimalist vaccine based on a rationally designed block copolymer (PEG-b-PC7a) showed the highest CTL activity when the pKa of the side chain of the cationic polymer segment was approximately 7 [107]. The effect was dependent on the activation of the STING pathway, but not on TLRs. Although the study reported on the encapsulation of peptide antigens, this system can potentially be used to encapsulate negatively charged antigen-encoding nucleic acids.

b. Polymers as a platform for functional conjugation

Polymers offer a versatile platform for the chemical modification and conjugation of cationic moieties, targeting signals, and small molecular weight adjuvants. Poly(lactic-co-glycolic acid) (PLGA) is an FDA-approved copolymer that is widely used in biomaterial applications owing to its biodegradability. Cetyl trimethylammonium bromide (CTAB)-modified PLG microspheres adsorb DNA on their surface via electrostatic interactions and have been used for vaccinations against HIV and HbV in mice [108,155]. Total IgG production was found to correlate with particle size, in which submicron particles induced the highest antibody production. Although the mechanism by which these cationic microspheres induce immune adjuvant activity has yet to be fully elucidated, DNA adsorption was found to play a key role in their efficient uptake by DCs, where coadministration of microspheres and DNA separately did not induce sufficient immunization [108]. A block copolymer based on linear PEI conjugated to PEG, installed with a DC-targeting peptide (sequence: FYPSYHSTPQRP), was used to prepare pDNA polyplexes. Uptake was significantly enhanced in the DC2.4 cell line, resulting in a higher efficiency of gene transfer [203].

Lynn et al. conjugated imiquimod or R848 (resiquimod), small molecules activating TLR7/8, on a polymer scaffold [42]. They found that by increasing the density of drug conjugation, the polymers form supramolecular assemblies in the submicron size range, which is pivotal for accumulation in draining lymph nodes to induce local immunization. In addition, particle formation of adjuvant restricted the inflammatory cytokine production to the local lymph following subcutaneous injection, whereas cytokine production in the systemic circulation was minimal. Reduction in systemic inflammation is a very crucial point to reduce systemic toxicity of adjuvants. HIV-related antigens were tethered to the particles to coadminister the adjuvant and antigen at the same target; however, there are no reports on the use of this system for nucleic acid-based vaccination. A similar approach was adopted by allowing resiquimod with a positive charge to bind to the negatively charged polymer adjuvant polyphosphazene via electrostatic interactions, forming a stable particulate system with dual adjuvant activity [204]. Cationic modification of polyphosphazene was carried out using imidazole and 2-dimethylaminoethylamino (DMAEA) side groups, conferring a cationic charge needed to condense pDNA [205,206]. Polyphosphazene is a promising polymer for pharmaceutical applications due to its hydrolytic degradability and the formation of nontoxic degradable products. Although the polyphosphazene mechanism of the adjuvant effect is poorly understood, it can be attributed to the formation of nonspecific ion interactions with TLRs 2, 3, 4, 5, 7, 8, and 9 that are overexpressed on engineered HEK293 cells [109,111]. In another report, polyphosphazene was found to trigger the local production of cytokines and chemokine CCL-2 at the site of injection [110].

### 3.2. Adjuvants Associated with Nucleic Acids

In addition to their use to express antigen proteins following vaccination, nucleic acids can be recognized by a wide range of PRRs both in the endolysosome or cytosol, giving rise to potent activation of innate immunity needed for vaccination. We summarized these receptors and the optimal ligand characteristics needed for binding in Table 2.

**Table 2:** Innate immune receptors sensing of nucleotides.

Location of PRR	Receptor	Ligand	Sequence Preference	Length Preference	Reference
Endo/lysosomal	TLR3	dsRNA	Low sequence preference	>45 nt	[207]
	TLR7	ssRNA	GU-rich sequence	N/A	[59]
	TLR8	ssRNA	GU-rich sequence	>20 nt	[208]
	TLR9	DNA	Unmethylated CpG	>20 nt	[209]
Cytosolic	RIG-I	dsRNA	5′ triphosphate at blunt dsRNA end	>20 nt	[210,211]
	MDA-5	dsRNA	N/A	>2000 nt	[212]

N/A: not available.

#### 3.2.1. Single-Stranded Structures

- mRNA

mRNA is inherently immunostimulatory, activating various cellular immune receptors in the endosome or cytosol [4,213]. The self-adjuvanted property of exogenously administered antigen-encoding mRNA is one of the main characteristics of mRNA vaccines. However, the influence of mRNA self-adjuvant activity on vaccine outcomes remains controversial. mRNA condensed in lipoplexes produced large amounts of type I IFNs after vaccination, which exerted an inhibitory effect on T cell response in the case of intramuscular and subcutaneous administration, but not intravenous vaccination [214,215]. Type I IFNs often hamper antigen expression, plausibly by the production of RNases and arresting protein translation, as part of their antiviral mechanisms. The chemical modification of mRNA is usually adapted to modulate its immunostimulatory activity and thus enhance antigen protein expression efficiency [216,217]. The effects of chemical modification are discussed in the section below. Single-stranded RNA (ssRNA), including unmodified mRNA, is a potent activator of endosomal TLR7 and -8, leading to the activation of the MyD88 pathway and the production of proinflammatory cytokines and type I IFNs [56]. Karikó et al. showed that mRNA can also activate TLR3 in endosomes, a receptor that is usually activated by double-stranded RNA (dsRNA) [218]. This could be attributed to the presence of secondary structures in mRNA that give rise to double-stranded regions, such as hairpins. TLR3 activation was observed both by mRNA released by necrotic cells and in vitro-transcribed mRNA. Inside the cytosol, mRNA can also activate the Nucleotide-binding oligomerization domain-containing protein 2 (NOD2) receptor, subsequently activating the type I IFN pathway [219].

Vaccination using naked unmodified mRNA is usually hampered by poor cellular internalization. Electroporation has been used for the ex vivo transfection of mRNA in DC-based vaccinations [220]. In a first-in-human trial in patients with advanced melanoma, mRNA-transfected DCs by electroporation were found to induce the recruitment of CD4+ and CD8+ T cells and demonstrated safety and survival benefits without the need for additional adjuvant [114]. In vivo, the ultrasound-guided percutaneous injection of naked mRNA into inguinal lymph nodes in patients was tested in melanoma patients in a phase 1 clinical trial [221]. The mechanism of mRNA uptake by dendritic cells in the lymph nodes was found to involve micropinocytosis [112]. Similarly, the intradermal administration of naked mRNA in mice was shown to be taken up by skin resident DCs by macropinocytosis, inducing the efficient priming of CD8+ T cells [113]. The intradermal injection of TAA-encoding naked mRNA has also been tested in clinical trials of melanoma patients [213]. However, recent trends favor the use of delivery systems to increase the transfection efficiency of mRNA and enhance its stability. Chahal et al. utilized self-adjuvanted mRNA formulated in adjuvant-free dendrimer nanoparticles for vaccination against H1N1 influenza, *Toxoplasma gondii*, and Ebola virus in mice, which resulted in both antibody and CD8+ immune responses and generated protective immunity by a single dose [222]. Although unmodified mRNA is self-adjuvanted, the level of immune activation can be insufficient and need to be augmented in some cases, which requires the addition of other adjuvants.

b. Effect of chemical modification on the mRNA immunostimulatory effect

**Figure 5:**
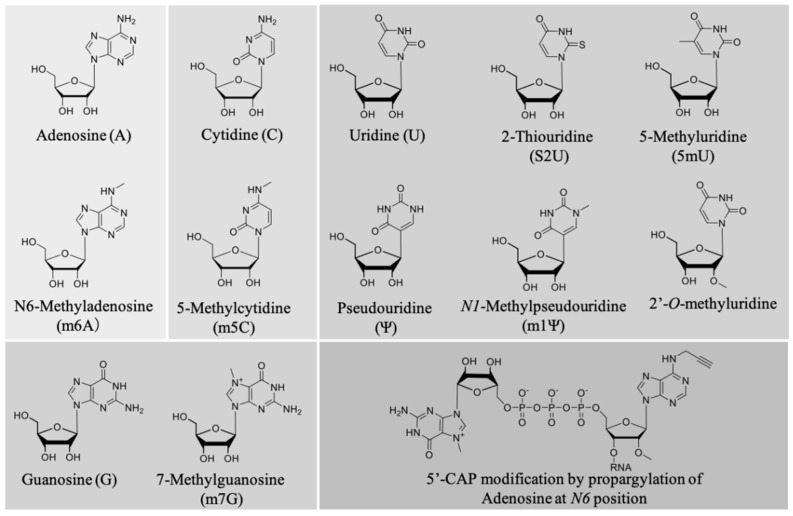
Commonly adapted chemical modifications in mRNA vaccines.

A pioneering work by Karikó et al. described the effect of nucleoside modification on the immunostimulatory effect of mRNA [223,224]. In vitro-transcribed mRNA was prepared in which one or two of the four nucleotide triphosphates (NTPs) were replaced by a corresponding naturally occurring nucleoside-modified NTP (Figure 5). The modification of A, C, and U NTPs, such as m5C, m5U, s2U, m6A, Ψ, or 2′-*O*-methyl-U modifications, generally suppressed the capacity of RNA to activate cytokine-generated DCs, as evidenced by reduced TNF-α and IL-12 production, as well as downregulating the expression of activation markers, such as CD80. Interestingly, only the modifications corresponding to UTP resulted in the ablation of blood-derived DC activation [223]. This highlights nucleoside modification as a powerful tool to modulate the magnitude of innate immune system activation depending on the desired vaccine outcomes. Notably, we previously screened the relationship between different types of nucleoside modification and mRNA translational activity, and found that mRNA nucleoside modification exerts both negative and positive effects on protein expression [216]. Thus, it is paramount to bear the balance between the adjuvant effect and translational activity of a given modified mRNA in mind for optimal vaccine design. While most of the mRNA chemical modifications adapted so far reduce mRNA immunogenicity, a very recent paper reported that 5′ cap modification by propargylation at position N6 of adenosine increased mRNA immunogenicity through the NF-κB signaling pathway, while maintaining translational activity [115]. This strategy has the potential to improve the effects of mRNA vaccines by chemical functionalization as a new adjuvant strategy. Figure 5 illustrates the commonly used chemical modifications of mRNA.

It is worth noting that mRNA vaccine delivery in noninflammatory settings is required for certain therapeutic applications. A recent report by Krienke et al. succeeded in transfecting splenic APCs using adjuvant-free liposomes encapsulating autoantigen-encoding mRNA to induce antigen-specific tolerance in mouse models of multiple sclerosis, an autoimmune disease of the brain. Mice immunized with 1 methylpseudouridine (m1Ψ) mRNA, encoding oligodendrocyte glycoprotein, showed significant protection against disease progression compared to unmodified mRNA [225].

#### 3.2.2. Double-Stranded Structures

- dsDNA

The main characteristic of nucleic acid vaccines is their ability to employ cell machinery to transcript/translate antigen peptides. To this end, several groups have described the use of engineered pDNA-co-expressing antigens and immunostimulatory motifs [118,119]. Luke et al. designed pDNA-expressing H5N1 influenza virus hemagglutinin as an antigen and an eRNA motif, which expresses immunostimulatory dsRNA by convergent transcription [119]. RNA polymerase III-transcribed RNA then exits the nucleus by exportin and presents a short dsRNA with 5′-PPP as a potent agonist of RIG-I, which subsequently upregulates the type I INF pathway. Prime-boost intramuscular vaccination in mice yielded a strong antibody response against H5N1 influenza virus in mice [119]. In another report, minimalized pDNA, namely nanoplasmids, coexpressing either Venezuelan equine encephalitis virus or Ebola virus glycoprotein and eRNA or CpG immunostimulatory motifs elicited a strong antibody response [118]. Nanoplasmids are smaller than traditional plasmids, allowing for improved uptake without the need for electroporation.

Bacterial extracts are well-known activators of the innate immune system. Unmethylated CpG dinucleotide repeats represent the molecular signature of the immunostimulatory signals derived from bacteria, which are required to activate TLR9 on B cells and plasmacytoid DCs [60]. Several types of synthetic unmethylated CpG oligodeoxynucleotide (ODN) motifs have been introduced as synthetic TLR9 agonists, which differ in the number of CG repeats on a phosphorothioate backbone [226]. CpG-based adjuvants have the capability to boost immunity in groups with reduced immune function, including the elderly, newborns, and immunosuppressed patients. Simian immunodeficiency virus (SIV)-infected macaques failed to mount any measurable antibody response when vaccinated against a hepatitis B vaccine; however, the addition of CpG ODN to the vaccine boosted considerable amounts of neutralizing antibodies [227]. Several CpG-based modalities have been tested for the treatment of cancers and infectious diseases in human clinical trials, with some being licensed for human use [228].

In pDNA vaccines, unmethylated CpG recognition by TLR9 was found to play a significant role in the induction of CD8+ T-cell responses. Additionally, LPS contaminants present in pDNA preparations could also induce CD8+ T cell priming via the MyD88-dependent pathway [116]. The presence of CpG in pDNA is often associated with decreased protein expression due to a decline in mRNA copy numbers rather than translational efficiency [229]. However, both the localization and precise sequence of CpG-containing regions are important for vaccination efficiency. In this regard, optimized pDNA with CpG motifs exhibited similar EGFP expression profiles in in vitro-transfected cells compared to their unmodified counterparts, and induced higher prophylactic activity when mice were challenged against melanoma cells due to their adjuvant effect [117]. While there are three major types of CpG motifs (types A, B, and C), all of them enhanced the vaccination effects of pDNA vaccine in different manners [230], with a combination of them further potentiating pDNA vaccines [231].

DNA nanotechnology allows for the formation of various supramolecular architectures of DNA. For example, Y-shaped oligodeoxynucleotides (Y-ODNs) were prepared using three CpG ODNs and induced a higher level of immunostimulation through cytokine release, such as TNF-α and IL-6, compared to conventional CpG ODNs [120]. Furthermore, Y-ODN bound to linear ODN (L-ODN) by complementary DNA sequences that also contain CpG on one arm, forms nanogels, improves the particle drainage distribution from the injection site, accumulates in draining lymph nodes, and induces higher immunostimulation. The coadministration of OVA with CpG nanogels enhanced antigen-specific antitumor activity in mice [121].

b. dsRNA

Mammalian cells can recognize specific structures of dsRNA as danger signals by TLR3 and other cytosolic receptors [62,232]. Synthetic long dsRNA analogs, such as Poly-IC and its derivatives, poly-IC12U and poly-ICLC, activate TLR3 and have been used to elicit Th1 immune profiles and enhance antibody and cell-mediated immunity [233]. Although the molecular weight of Poly-IC varies between 100 and 325 kD depending on the supplier, its large polyanion structure allows for electrostatic interactions with other materials for the formation of the size-controlled particles needed for vaccination [234,235]. Nanoparticles prepared by the ionic gelation technique from chitosan and poly-IC with a net positive charge were used to adsorb negatively charged antigens [122]. These polymer-based vaccines have the advantage of retaining stability after freeze-drying, which expands their industrial potential. The layer-by-layer coating of live Bacille Calmette–Guerin (BCG) using repeating layers of poly-IC and chitosan enhanced and modulated the responses to the BCG vaccine [236]. Poly-IC is internalized into macrophages and other cells, such as fibroblasts and epithelial cells, by receptor-mediated endocytosis through interaction with scavenger receptors and other receptors, such as Mal-1, which can exert a positive effect on cellular uptake and subsequent type I INF responses [237,238].

**Figure 6:**
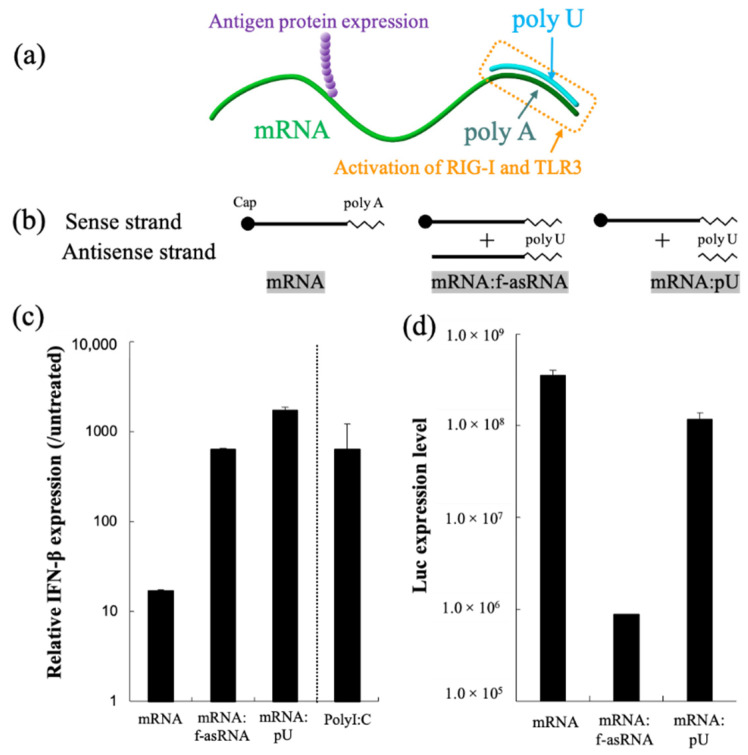
RNA hybridization as a novel technology to induce a specific adjuvant effect. (**a**) Partially double-stranded mRNA with poly U hybridized to poly A simultaneously expresses antigen protein and stimulates RIG-I and TLR3. (**b**–**d**) Functionalities of partially double-stranded mRNA. (**b**) Luciferase mRNA without hybridization(mRNA), mRNA hybridized with full-length antisense RNA (mRNA:f-asRNA), and mRNA hybridized with poly U (mRNA:pU). (**c**) Immunostimulatory property. (**d**) Translational efficiency. After luciferase mRNA introduction to cultured dendritic cells, levels of interferon β transcript (**c**) and Luc protein expression were quantified. Reproduced with permission from [123], Elsevier, 2018.

mRNA hybridization using short oligonucleotides, creating partially double-stranded RNA, represents a versatile methodology that functionalizes mRNA. In our previous report, we attached a short complementary strand of RNA to the poly A segment of mRNA, creating a short dsRNA required for the activation of RIG-I and TLR3, and found that the translational activity of mRNA remained high (Figure 6) [123]. A single injection of partially double-stranded mRNA in the inguinal lymph node of mice resulted in a stronger production of antibody titers and reactive T cells compared to unmodified mRNA. Additionally, structuring mRNA by hybridization could be a promising technique to control the particle size required for particle drainage following vaccine administration. In a separate report, we bundled mRNA strands using short oligonucleotides, which allowed us to enhance the stability of mRNA against nuclease-mediated degradation. These sub-100 nm spherical particles released mRNA inside the cell using endogenous mRNA unwinding mechanisms, and therefore exhibited efficient protein expression when injected into mouse brain [239].

Self-amplifying (SAM) RNA is a long RNA that encodes both antigens and replicases, which are required to produce multiple antigen copies [240]. This system provides not only prolonged expression of antigen, but also induces strong immunostimulatory effects owing to formation of the dsRNA structure during RNA self-amplification, which interacts with multiple PRRs. SAM RNA activates a type I IFN through endosomal sensing via TLRs 3/7/8, as well as cytosolic sensing via MDA5, RIG-I, and possibly unknown pathways [241]. Self-adjuvanted RNActive vaccine technology based on SAM RNA has emerged as a promising vaccine technology, inducing strong and balanced immune responses comprising humoral and cellular responses. However, due to prolonged and uncontrollable immunostimulation induced by SAM RNA, the rate of side effects of this strategy is high and is mainly characterized by reactogenic effects [124].

## 4. Conclusions

Vaccines are designed for mass administration in response to infectious disease pandemics as well as for individual administration when targeting cancer neoantigens. In both cases, whether the vaccine is intended for use for a single patient or millions of patients, simple formulations that are easy to design, produce, and characterize are necessary to reduce manufacturing complexity and ensure quick on-demand production. The incorporation of multifunctional immunoadjuvants into pDNA/mRNA vaccines represents a promising approach to enable minimalist vaccines with a controlled induction of the innate immune system and a desired protective immune response, but a reduced rate of serious side effects. Although the level of multifunctionality varies among the diverse classes of immunoadjuvants reviewed in this article, ionizable and cationic materials represent the most multifunctional immunoadjuvants to date. Furthermore, the mechanism of immunoadjuvant activity of many of the materials appears to be diverse and nonspecific, which calls for the design of more specific multifunctional immunoadjuvants to be tailored for specific vaccine effects in the future.

## Data Availability

Not applicable.
